# S-Phase Favours Notch Cell Responsiveness in the *Drosophila* Bristle Lineage

**DOI:** 10.1371/journal.pone.0003646

**Published:** 2008-11-05

**Authors:** Sylvie Remaud, Agnès Audibert, Michel Gho

**Affiliations:** 1 Université Pierre et Marie Curie, UMR 7622, Paris, France; 2 CNRS, UMR 7622, Paris, France; Ordway Research Institute, United States of America

## Abstract

We have studied cell sensitivity to Notch pathway signalling throughout the cell cycle. As model system, we used the *Drosophila* bristle lineage where at each division N plays a crucial role in fate determination. Using *in vivo* imaging, we followed this lineage and activated the N-pathway at different moments of the secondary precursor cell cycle. We show that cells are more susceptible to respond to N-signalling during the S-phase. Thus, the period of heightened sensitivity coincided with the period of the S-phase. More importantly, modifications of S-phase temporality induced corresponding changes in the period of the cell's reactivity to N-activation. Moreover, S-phase abolition was correlated with a decrease in the expression of *tramtrack*, a downstream N-target gene. Finally, N cell responsiveness was modified after changes in chromatin packaging. We suggest that high-order chromatin structures associated with the S-phase create favourable conditions that increase the efficiency of the transcriptional machinery with respect to N-target genes.

## Introduction

How cell fate signalling pathways interact with the cellular machinery that controls the cell cycle is not yet clear. More specifically, little is known about how cell sensitivity to fate signalling mechanisms fluctuates over different phases of the cell cycle. Here we analyse whether cell responsiveness to Notch (N) signalling, an essential developmental regulatory pathway, varies during successive phases of the cell cycle.

N-signalling is essential to pattern formation and cell fate determination in many developmental systems [1]–[3]. The interaction between Notch receptors and their ligands (Serrate and Delta in *Drosophila*) triggers N signalling by way of evolutionarily conserved intramembrane cleavage events [3]. These events allow the translocation of the cytoplasmic domain of the receptor into the nucleus where it participates, along with other factors such as the DNA-binding protein Suppressor of Hairless (Su(H)), in the formation of a transcriptional activator complex [4]. Cell fate diversity mechanisms involving N-signalling are implicated in both lateral inhibition, an essential process in which one cell is selected from an equivalent cell group after a mutual exchange of signals, as well as cell fate induction, in which a competent cell is committed to its fate after unidirectional signalisation.

A clear relationship between cell cycle and N-mediated cell determination processes has been observed in asymmetric cell divisions that take place during *Drosophila* neurogenesis [5]. Here, cells involved in N-signalling are mitotically related and the direction of the N-signalling is controlled by differential segregation of N-modulators during mitosis, such as Numb and Neuralized (Neur) (see for review [6], [7]). Nevertheless, the relationship between cell cycle and cell determination does not seem to be limited to the asymmetric segregation of fate determinants during mitosis. The progression through the S-phase also appears to be required for the acquisition of determined cell identities. Thus, after self-renewed division of the *Drosophila* 2–7 neuroblast, the smallest daughter cell resulting from this division acquires a Ganglion Mother Cell fate only if it undergoes DNA replication [8]. Moreover, during vulva formation in *C. elegans*, N-signalling occurs only during the S-phase, suggesting some degree of dependency [9]. These observations are consistent with the idea advanced thirty years ago that DNA-replication renders regions of the genome available for transcription that are normally not accessible in other phases of the cell cycle [10].

**Figure 1:**
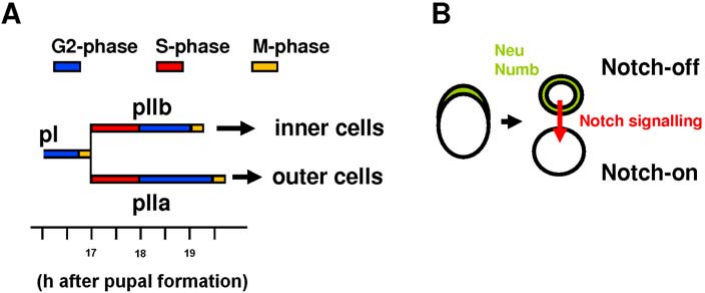
The bristle lineage. (A) pI cells divide giving rise to two secondary precursor cells, pIIb and pIIa. The former, after two rounds of divisions, produces the inner cells and the latter, after one round of division, the outer cells. The corresponding cell cycle phases are shown in colour (modified from [19]). (B) Asymmetric cell division of pI. The differential segregation of Numb and Neuralized (Neu) during pI division triggers unidirectional Notch-signalling to the posterior cell. As such, the anterior sister cell becomes the ligand sender (N-off, pIIb) and the posterior cell the signal receiver (N-on, pIIa).

Many studies have been performed on *Drosophila* to analyze cell cycle machinery, cell fate acquisition and signalling pathways partly because of the low level of genetic redundancy exhibited by this organism. In particular, the cell lineage leading to the formation of mechanosensory microchaete (hereafter referred to as bristles) in *Drosophila* has become an excellent model system to analyse the relationship between cell division and cell determination [11]. During the formation of these bristles (around 12 hours After Pupal Formation, APF), N-mediated lateral inhibition is involved in selecting the primary precursor cell (pI) from a cluster of equivalent cells, called a proneural cluster. Down-regulation of the N-pathway during this period results in a neurogenic phenotype characterized by the formation of extra bristles [12]. Once determined, pI initiates a rapid sequence of four asymmetric cell divisions giving rise to five different cells (see Fig. 1A) [13]. At each division, one daughter cell (N-off) acts as a N ligand-producer and the other (N-on) as a N signal-receiver [14], [15]. The bias in the activation of the N-pathway is assured by the stereotyped segregation of Numb and Neuralized in one cell which blocks the N-receptor and promotes N-signalling respectively [16], [17] (see Fig. 1B). During the first round of division (at about seventeen hours APF), pI divides, roughly parallel to the midline, giving rise to two secondary precursor cells. Numb and Neuralized are inherited by the anteriorly located secondary precursor cell that then acquires a pIIb fate (it becomes the N-off cell and corresponds to the N-signal sender cell of the pair). The posteriorly located cell, the pIIb sister cell, acquires a pIIa identity (it becomes the N-on cell and corresponds to the N-receiver cell). During the next division (around nineteen hours APF), the pIIb cell generates a tertiary precursor cell, pIIIb, and a glial cell that enters apoptosis shortly after birth. The subsequent division of pIIIb produces the inner cells of the organ (the sheath cell and the neurone). The division of pIIa leads to the formation of the outer cells of the organ (the shaft and socket cells) [13], [18] (Fig. 1A). The essential role of the Notch pathway in cell fate acquisition in this organ is demonstrated by the fact that, in absence of Notch activity, both precursor cells acquire a pIIb fate and the resulting organ is composed exclusively of inner cells. Reciprocally, organs composed exclusively of outer cells are obtained after ectopic activation of the N-pathway [12].

Previous analysis of cell cycle phases in each cell of the bristle lineage revealed that the G1-phase was essentially absent in these cells [19]. Thus, in secondary precursor cells, the S-phase begins directly after birth and lasts for about one hour. It is followed by the G2-phase that continues until the next round of division (Fig. 1A). A precise knowledge of the timing of these phases allows us to study whether cells express a differential sensitivity to the Notch pathway during S and G2 cell cycle phases. Here we show, using an *in vivo* approach, that cells from the *Drosophila* bristle lineage are more sensitive to N-activation during the S-phase than during the G2-phase. In addition, this differential N-responsiveness of bristle cells could be modified under experimental conditions that affected the activity of chromatin-remodelling factors. Thus, we suggest that progression through the S-phase overrides local high-order chromatin folding structures in genomic regions essential to implement the response to N-pathway activation.

## Results

We used *in vivo* imaging techniques to analyse fluctuations in bristle lineage cell responsiveness to N-pathway activation. This pathway was activated at precise times of the cell cycle of the two secondary precursor cells of the lineage, pIIa and pIIb. To visualize bristle lineage cells, we expressed YFP-tagged histone2B proteins (hereafter H2B::YFP, [20]) using the UAS-Gal4 binary expression system [21]. The *neuralized^P72^*-Gal4 (*neur>*) line was used to specifically express the constructions in pI and its progeny. Both secondary precursor cells were identified as the daughter cells resulting from the division of pI cells. After completion of the lineage, the presence of inner and/or outer cells was used to monitor the identity acquired by the secondary precursor cells and as such to measure the sensitivity of these cells to the N pathway. The thermosensitive *N^ts-1^* allele was used to enable endogenous N-pathway activation after a temperature shift from 30°C to 18°C. The overexpression of the active form of the N-receptor (N^intra^) driven by a heat-shock promotor was used to ectopically trigger the N-pathway after a temperature pulse (HS) to 37°C for 10 min.

### The kinetics of the cell response under N^ts-1^ and N^intra^ conditions was fast enough to activate the N-pathway during the cell cycle phase studied

In a series of control experiments, we ascertained that the kinetic of the N-response triggered under *N^ts-1^* and *N^intra^* conditions were compatible with the timing of phases of the cell cycle.

In the *N^ts-1^* strain, the N receptor behaves as a wild-type at 18°C (permissive temperature) and as a strong hypomorph at 30°C (restrictive temperature). The kinetics of the *N^ts-1^* allele inactivation was measured by applying temperature shifts from 18°C to 30°C at precise times before and after pI division (Supporting data Fig. S1A). The formation of sensory organs composed exclusively of inner cells was used to assess the delay required to block N-function at 30°C. This analysis revealed that the N-function was blocked when temperature was shifted prior to and between 0 and 15 min after pI division. More interestingly, temperature shifts applied later than 15 min following pI division were ineffective in blocking the N-response. These findings show that the *N^ts-1^* receptor is rapidly rendered non-functional after 15 minutes at a restrictive temperature. As such, when experimental conditions required an N-off state, *N^ts-1^* pupae were shifted to 30°C for at least one hour before beginning data acquisition.

We analyzed the kinetics of *N^ts-1^* activation by applying temperature pulses of variable duration (to 18°C) to *N^ts-1^* pupae maintained at 30°C (see Supporting data Fig. S1B). The formation of normal sensory organs composed of both inner and outer cells (proof that N-on cells were formed) was used to assess the time required for *N^ts-1^* receptors to recover their function at 18°C. We observed that N-on cells were present following temperature pulses lasting longer than 15 minutes (Supporting data S1B). This shows that *N^ts-1^* receptors recover their function within 15 min at 18°C. Taken together, these data suggest that the activation and inactivation kinetics of the *N^ts-1^* receptor are similar, around 15 minutes.

In the *HS-N^intra^* strain, we analyzed the kinetic of N-signalling by following, by immunodetection, the accumulation of N^intra^ induced after a HS. N^intra^ was clearly detected in the nucleus 20 minutes after HS. This nuclear signal reached its peak about 30 min post-HS and remained visible for around 2 hours (Supporting data, Fig. S2). Moreover, the time-course of N^intra^ accumulation was the same whether the HS was applied during the first hour (during S-phase) or the second hour (during G2-phase) of the secondary-precursor-cell's life (Fig. S2). A similar time course of N^intra^ accumulation following a HS has been obtained in other systems [22], [23].

These data show that the N pathway is either functional or activated after a delay of approximately 15 or 20 minutes when the *N^ts-1^* allele or the *HS-N^intra^* construction were used respectively. This period is sufficiently short to enable us to analyse the cell response elicited by N-activation as a function of different cell cycle phases, since each phase lasts for one hour or more.

### Cells are most receptive to endogenous Notch pathway activation during their first hour of life

**Figure 2:**
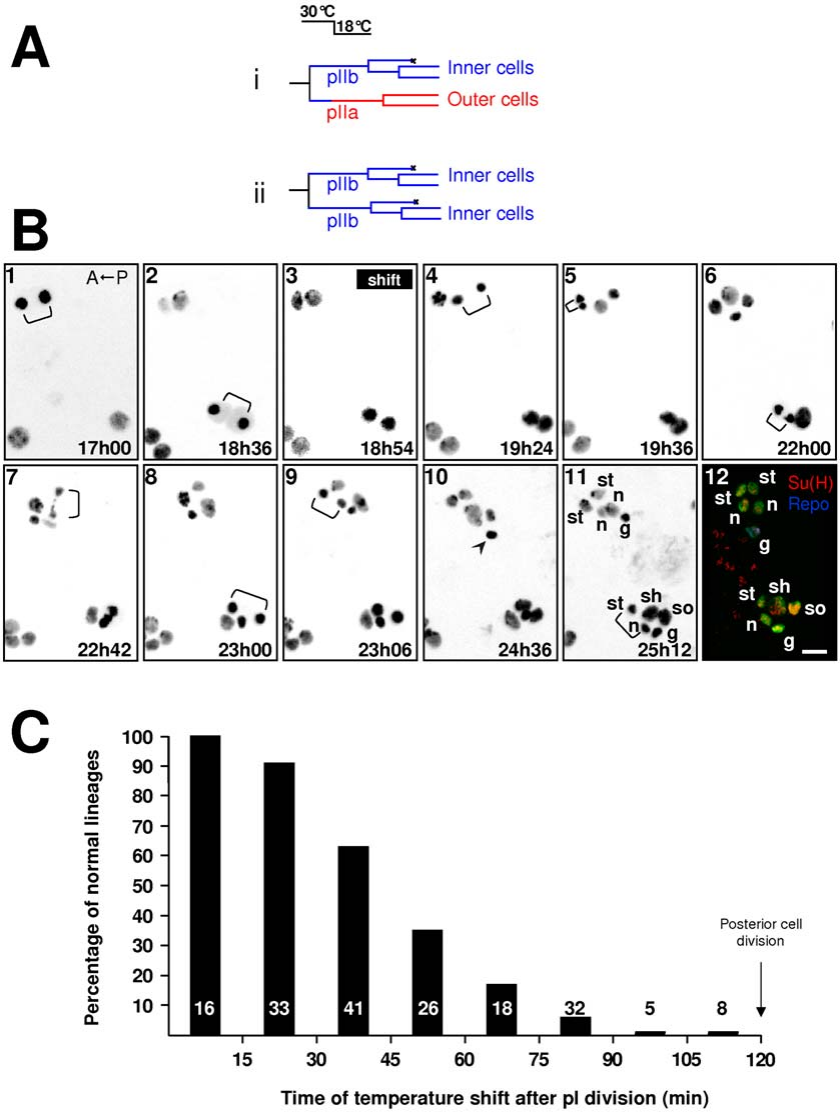
Cells are more receptive to endogenous N-pathway activation during their first hour of life. The acquisition of a pIIa identity was used as an index of Notch pathway activation in the posterior secondary precursor cell. A *N^ts^/Y; neu>H2B::YFP* pupae imaged *in vivo* was temperature shifted from 30°C to 18°C at a given moment after pI division enabling the N-receptor to be endogenously activated. A) Lineages expected if the posterior secondary precursor cell responded or not to Notch activation (i and ii respectively). (B) Representative frames from an *in vivo* recording followed by immunostaining of two microchaete lineages. Anterior is on the left. The temperature shift was applied at 18h54 after pupal formation (APF). Brackets indicate cell divisions. Time APF is shown at the bottom right of each frame. At 18°C, development proceeds half as quickly as at 25°C. The arrowhead (frame 10) indicates apoptosis of the glial cell. In frame 12, bristle lineage cells are revealed by anti-GFP antibody (green). Su(H) and Repo immunoreactivities were used to identify socket (so) and glial (g) cells respectively (yellow and blue). The other cell identities have been ascribed based on the characteristics of their divisions recorded *in vivo*, n: neuron, st: sheath, sh: shaft cell. Each image results from the merge of 5 horizontal optical sections. Note that when the temperature shift was applied 1h54 after the pI division (upper cluster), the resulting organ was formed exclusively by inner cells. In contrast, when applied 0h18 after the pI division (lower cluster), a normal organ resulted. Scale bar: 5 µm. (C) Percentage of normal sensory organs observed when the temperature shift was applied at different moments after pI division (abscissa). Note that normal sensory organs were observed when the temperature shift was applied during the first 45 min of the secondary precursor cells life. In this and other histograms, the numbers at the base of the bars indicate the number of sensory clusters analyzed.

In order to determine a time-frame during which posterior secondary cells are most sensitive to N-signalling, we maintained *N^ts-1^* pupae at 30°C for at least 1 hour prior to the experiment to create an “N-off” condition. Pupae were then imaged *in vivo* and at a given time following pI division, they were shifted to 18°C in order to permit endogenous activation of the Notch pathway (Fig. 2A). If the temperature shift falls during the period of heightened N-receptivity, the N pathway will be activated in the posterior secondary precursor cell by a signal coming from its anterior sister cell as in the wild-type situation. As a result the former will acquire a pIIa identity and the latter a pIIb identity thus resulting in a normal sensory organ (Fig. 2A, upper lineage). If, on the other hand, the N cell response is not implemented in the posterior precursor cell, both secondary precursor cells will acquire a N-off pIIb identity and the organ will be composed exclusively of inner cells (Fig. 2A, bottom lineage). Thus, the timing of the temperature shift that allows the formation of normal sensory organs is related to the period during which cells are receptive to the N-signal. These results are illustrated in Fig. 2B where we show representative frames from time lapse imaging of two clusters in *N^ts-1^; neur> H2B::YFP* living pupae (frames 1–11) combined with immunostaining (frame 12) (see also Supporting data, Movie S1). In this time-lapse recording, the upper cluster experienced the temperature shift at 1h54 after pI division (a late shift) and the lower cluster at 0h18 after pI division (early shift). In the upper cluster, both pI daughter cells behaved similarly and underwent two rounds of divisions (bracket in frames 4 and 7 and in frames 5 and 9 respectively). In addition, the resulting cells harboured small nuclei typical of inner cells (see frame 11). These observations show that, for late temperature shifts, no N response was triggered in the posterior cell and it behaved as a pIIb cell. Conversely, for early temperature shifts (lower cluster), the posterior cell divided only once (bracket in frame 8) instead of twice as did its anterior sister cell (bracket in frame 6 and 11). The size of the nuclei in the resulting cells differed, with two large nuclei for the posterior daughter cells and three small nuclei for the anterior daughter cells (frame 11). Taken together, these observations suggest that this lower cluster harbours a normal set of cells and that the N response was indeed triggered in the posterior cell, which then acquired a pIIa identity.

We confirmed these observations by immunodetection performed after both cell lineages were complete (8 hours after changing to the permissive temperature). We identified socket cells by the characteristic strong accumulation of Su(H) and glial cells by the expression of Repo [14], [18]. As shown in Fig 2B frame 12, the lower cluster contains outer cells (as shown by the presence of a Su(H) positive socket cell, yellow coloured cell). In this particular case, the glial cell was too immature for Repo detection. In contrast, the upper cluster was formed exclusively by inner cells (absence of Su(H) positive cells). In addition, two cells in this cluster behave as glial cells since one accumulates the glial specific marker, Repo (blue cell in frame 12 in Fig. 2B) [24] and the other enters apoptosis (the cell with a condensed nucleus in frame 10, arrowhead). We performed similar experiments using other cell markers and obtained the same results (see Supporting Fig S3). Using this combined technique of time-lapse imaging and immunodetection, 190 bristle lineages from at least 20 pupae were analysed. The results are summarized in Figure 2C in which the percentage of posterior secondary precursor cells acquiring a pIIa identity (in other words, the probability that these cells implemented a N-response) is plotted against the time of the temperature shift after pI division. We observe that more than 50% of the cells responded to endogenous N-activation when the temperature shift occurred within 45 minutes following pI division. After this time, the proportion of cells that acquire a pIIa identity diminished progressively and moreover cells did not respond at all if the shift occurred within the 30 minutes preceding the next division. These results show that the relationship between the cell's ability to generate a response after endogenous activation of the N-pathway and the time at which the N-response is implemented is not linear. Indeed, the probability of acquiring a pIIa identity is more important (>0.5) when the Notch receptor is able to respond within the first hour of life.

### Cells are more susceptible to respond to ectopic Notch pathway activation during their first hour of life

**Figure 3:**
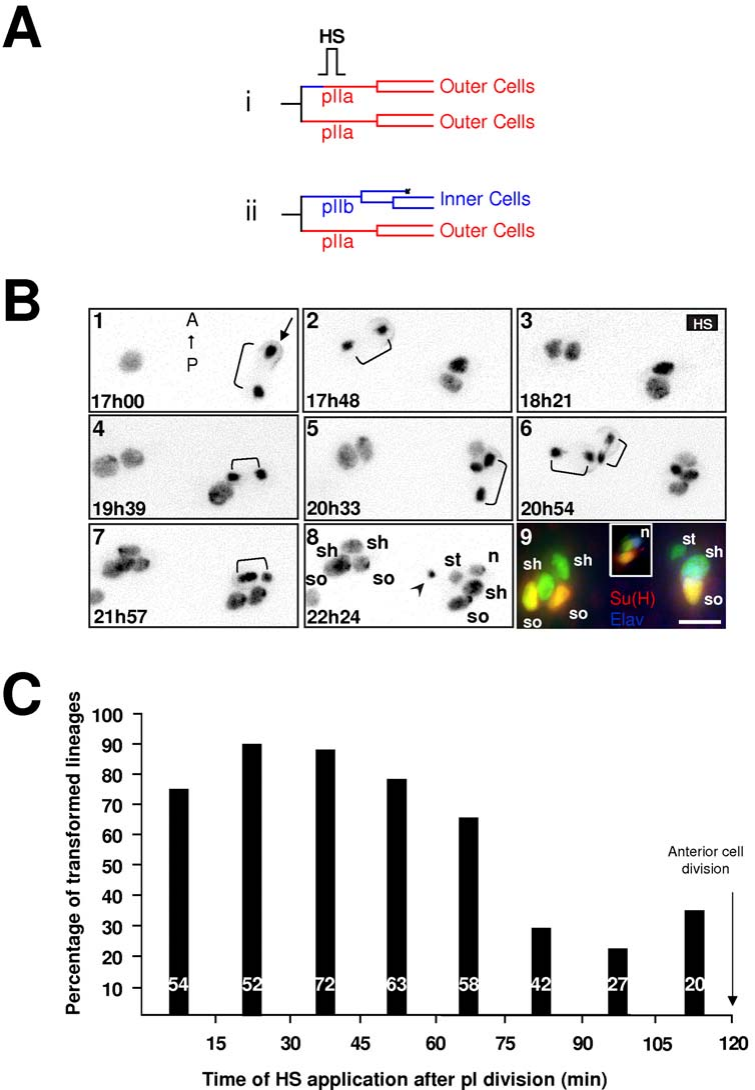
Cells are more receptive to ectopic N-pathway activation during their first hour of life. A *hs-N^intra^* pupae imaged *in vivo* was heat shocked (HS, 10 min at 37°C) at a given moment after pI division in order to ectopically activate the N-pathway in the anterior secondary precursor cell. (A) Lineages expected if the anterior secondary precursor cell responded or not to Notch activation (i and ii respectively). (B) Representative frames from an *in vivo* recording of two microchaete lineages of an *hsN^intra^; neu>PON::GFP, H2B::YFP* pupae in which a HS was applied between 18h16 to 18h26 APF. Anterior is on the top. Time APF is shown at the bottom left of each frame. The PON::GFP crescent was used to distinguish the polarity of each cell division (indicated only in frame 1, arrow). Brackets show cell divisions. The arrowhead in frame 8 indicates apoptosis of the glial cell. Each frame results from the merge of 7 horizontal optical sections. Frame 9 shows the final immunodetection. Su(H) in red, Elav in blue, sensory cells in green. The inset in frame 9 shows a lateral view of the right cluster highlighting the nucleus (n). Note that a normal sensory organ was generated when the HS was applied long after pI division (right cluster) whereas a sensory organ composed by outer cells exclusively is formed for earlier HS (left cluster). so, socket; sh, shaft; st: sheath, g: glial cell cells, n; neurone. Scale bar: 5 µm. (C) Percentage of sensory organs harboring two socket cells when the temperature shift was applied at different times after pI division (abscissa). Note that transformed sensory organs were observed when the HS was applied principally during the first hour of secondary precursor cells life.

The enhanced responsiveness to N-pathway activation during the first hour of life may be due to a higher receptivity of cells during this period. To test this possibility, we studied the temporal window in which the anterior cell responds to a direct ligand-independent activation of the N-pathway by overexpressing the active form of the N-receptor (N^intra^). To activate the N-pathway at defined moments after pI division, *hs-N^intra^* pupae were heat shocked under visual control (Fig. 3). We then observed whether or not the anterior cell (normally an N-off cell) transformed into pIIa (Fig. 3Ai). Two representative clusters in *hs-N^intra^; neur> H2B::YFP* living pupae are shown in Fig. 3B (see Supporting data, Movie S2) recorded with time-lapse imaging combined with immunostaining. The N-pathway was activated (HS at frame 3) 30 min after pI division for the left cluster (early activation) and 1h18 after pI division for the right cluster (late activation). When the N-pathway was activated early, the anterior cell divided once and almost simultaneously with the posterior cell (brackets in frame 6). Moreover, all resulting cells harboured large nuclei (see frame 8). This suggests that the anterior cell was transformed and acquired a pIIa identity. This was confirmed by immuno-detection (frame 9) showing that the left cluster was formed by four outer cells: two Su(H) positive socket cells (yellow cells) and two shaft cells (green sibling cells), showing that the organ originated from two pIIa cells. In contrast, when the N pathway was activated late, the anterior cell behaved as a normal pIIb cell and underwent two rounds of divisions (bracket in frames 4 and 7) instead of only one for its posterior sister cell (bracket in frame 5). As in a normal lineage, three out of the five resulting cells contained a small nucleus and one of these cells entered apoptosis (arrowhead in frame 8). The presence of a normal set of cells in the right cluster was confirmed by immunodetection (frame 9, right cluster). Among the final four cells present in this cluster one was a Su(H) positive socket cell (yellow cell) and one a neuron (blue cell, the inset shows a z-reconstitution to illustrate the nucleus that is not situated in the same plane as the other cells). The results obtained from 390 clusters (at least 25 pupae) are summarised in Fig. 3C in which the proportion of anterior cells acquiring a pIIa identity is plotted as a function of the time of heat-shock. Our results show that cells pass from a state of high responsiveness to N-pathway activation (up to 50%) during the first 60 minutes of life to a low, no null, responsiveness state (under 50%) with a period of transition around 60 to 75 minutes after birth. Statistical analysis confirms these observations since values obtained between 0 to 60 min, 60 to 75 min and 75 to 120 min APF after pI division were significantly different from each other (p<0.05 Chi-square Test and Bonferroni correction).

Similar results were obtained when cell transformation from an N-off to an N-on identity was monitored by the presence of Tramtrack (Ttk), a non-neuronal fate determinant that acts downstream of the N-pathway [16], [25], [26]. Ttk normally accumulates in pIIa prior to its division and was used as a marker of pIIa identity (control in Supporting data, Fig. S4). We observed that a high proportion of anterior cells became Ttk-positive only when the N-pathway was activated by a *hs-N^intra^* pulse during the first 45 minutes after birth.

Taken together, these data support the idea that the enhanced responsiveness to N-pathway activation during the first hour of life is related to a cell's intrinsically high degree of sensitivity to N-signalling.

### The temporal dependence on Notch was not related to the time required to implement the Notch cellular response

It is likely that a cell's probability of becoming an N-on cell following N activation is governed in part by the proximity of N-activation to the subsequent cell division. In other words, the closer the moment of N-activation is to the next cell division, the lower the probability of the cell becoming N-on. Thus, the fact that cells are less sensitive to N-activation during their second hour of life may simply reflect a critical period necessary to implement the N-response. In order to study this possibility, we activated the N-pathway at different times after pI division and asked whether the probability to respond to N-activation depended on the moment of the heat-shock or on the time-lapse after the heat-shock. We predicted that if the enhanced sensitivity to N-activation depended on the delay after N-activation, the probability to respond to N-activation would increase with longer delays.

**Figure 4:**
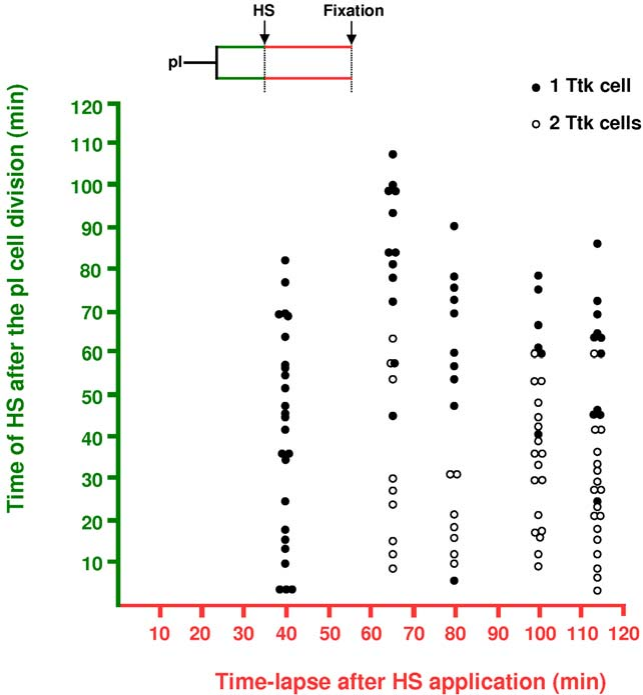
The probability to respond to the N pathway was related to the moment of N-expression rather than to the period required to implement the cellular response to Notch. Correlation between the moment of the N^intra^ pulse (via a HS) after pI division (ordinate) and the time-lapse following N-activation (abscissa). The ectopic expression of Ttk in secondary anterior precursors was used to identify cell fate transformation (empty circles, both secondary precursor cells were Ttk positive). Filled circles correspond to no transformation (only one Ttk positive secondary precursor cell). Also note that the minimal time required to detect Ttk in the anterior cell was about 60 min. Note also that the transformation efficiency is higher when N^intra^ pulses were applied during the first hour of life (empty dots are concentrated in the bottom half) independently of the time lapse after HS. *hsN^intra^; neu>H2B::YFP* pupae.

The N-pathway was activated via HS at different moments after pI division and the nota were dissected out at either 40, 65, 80, 100 or 115 min post-HS. The ectopic accumulation of Ttk in the anterior cell was used to identify cell transformation to an N-on pIIa identity (Fig. 4, empty dots). We detected Ttk in the anterior cell only after 60 minutes or more following HS. Thus, with Ttk detection used as a cellular marker, 60 minutes was the minimal time required to identify a cell transformation after ectopic N-activation. Interestingly, at 115 minutes after HS (almost twice the minimal time required to detect Ttk), 11 out of the 29 anterior cells studied were still not transformed (filled circles). Thus, after the initial 60 min required for the expression of *ttk*, no correlation was observed between the probability of cell transformation and the time-lapse following N-activation. In contrast, a correlation was clearly apparent between cell transformation and the moment of the heat shock. Nearly all transformations were induced when HS were applied between 0 and 60 minutes after pI division (Fig. 4, bottom half of the plot). These observations show that fate transformation is related to the moment of N-expression rather than to the duration of the period following N-activation. Thus, it seems that during their first hour of life, cells pass through a particular state that allows them to be more apt to respond to N-pathway activation.

Consistent with the notion that cells are more sensitive to N during the first hour of life, the critical period during which the N-receptor inhibitor Numb can affect the N-dependent response occurred within this same period of time. Using a *HS-numb* line, we overexpressed Numb at defined moments during the life of the secondary precursor cells and we determined the proportion of transformed organs harbouring exclusively inner cells as a function of the time of HS application (Supporting data Fig. S5). Overexpression of Numb effectively blocks (>50%) the N-response when the HS was applied during the first 30 minutes of life while HS applied later had no effect. These data show that the N-on response is implemented during the first 30 minutes of life, after which cells seem to be committed and are no longer affected by Numb. In accordance with these results, endogenous Numb, which is asymmetrically distributed during pI division, can be readily immunodetected in the anterior N-off cell during the first 10 minutes after pI division (not shown).

### Cell competence to Notch activation depends on the S-phase

**Figure 5:**
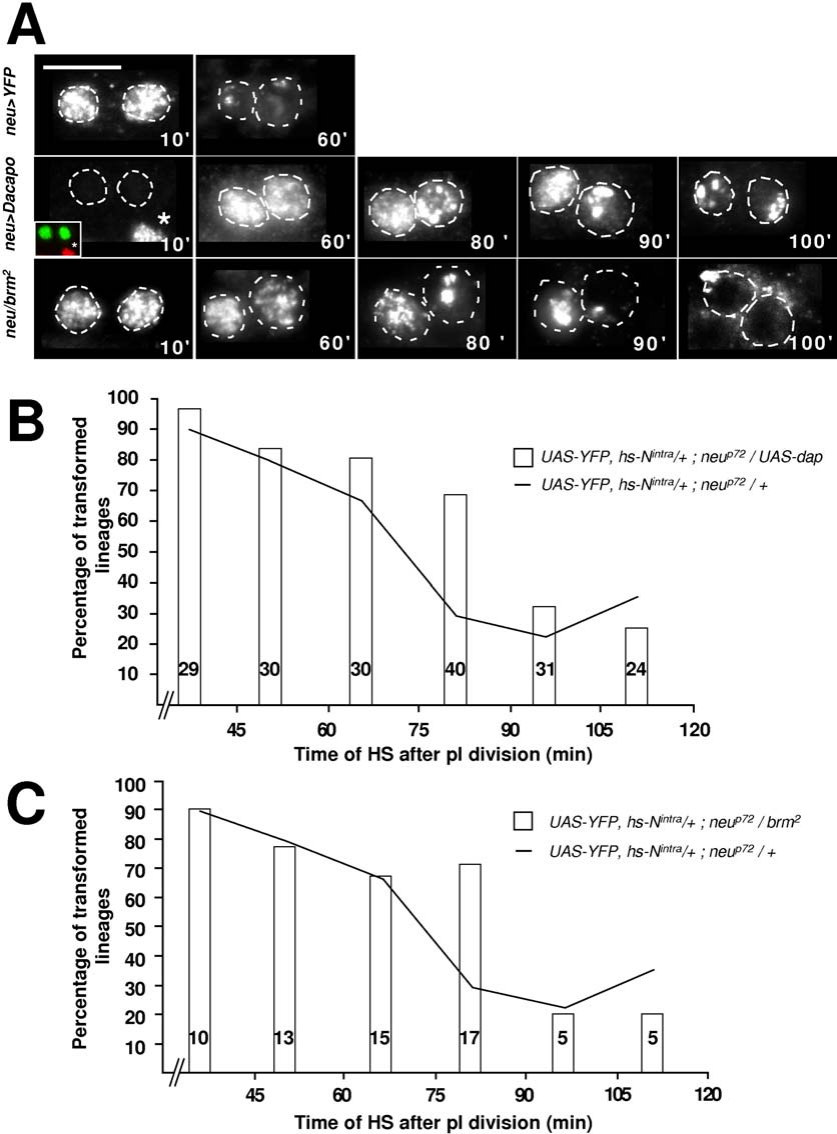
The timing of the response to Notch is correlated with the S-phase. (A) The duration of the S-phase was monitored by combining time-lapse imaging and BrdU incorporation. The age of clusters after pI division is indicated at the bottom right of each frame (in min). Anterior is on the left. Note that Dacapo overexpression shifted the S-phase while, in *brm^2^* heterozygous flies, the S-phase was lengthened. The inset in the 10' frame in Dap overexpression images shows BrdU incorporation (in red) in epithelial cells (asterisk) while there is no incorporation in the secondary precursor cells (in green). Scale bar: 5 µm. (B–C) Histograms show the percentage of transformed sensory organs (resulting from the formation of two pIIa cells) as a function of the moment of N activation (abscissa) in a *Dap* overexpression background (B) and in a *brm^2^* heterozygous background (C). Control data (hs-N^intra^ pupae alone) were collected throughout all experiments. Since we never saw any significant differences in these data, they were pooled and are presented in (C) and (D) as a continuous line. Note that in both cases the transformation was significantly higher when a N^intra^ pulse was applied between 75 and 90 min after pI division.

Our previous study has revealed that the G1-phase is absent in secondary precursor cells [19]. Thus, the S-phase starts during pI telophase and lasts for about one hour (see Fig 1A). We hypothesize that the enhanced sensitivity to N-activation during the first hour of life could be related to the DNA replication phase. To test this possibility, we modified the timing of the S-phase in secondary precursor cells and we analysed whether or not the temporal response to N-activation changed accordingly. In order to delay the entry into the S-phase, we overexpressed the CDK inhibitor Dacapo (Dap,[27], [28]) specifically in the bristle cells using the *neu-Gal4* driver. In control conditions, full BrdU incorporation was observed at 10 min after pI division and the punctuate incorporation of BrdU associated with the late replication of heterochromatin was observed at 60 min after pI division (Fig 5A, *neu>YFP*). Under conditions of Dap overexpression, secondary precursor cells did not incorporate BrdU during the first 20 minutes after birth (see 10' column in Fig. 5A, *neu>dap*). In addition, the punctuate incorporation of BrdU was not observed until 100 min after birth (Fig. 5A). Thus, after Dap overexpression, the S-phase in secondary precursor cells was delayed by around 20 minutes. Associated with this 20 min delay, the enhanced response window to N-activation was prolonged (Fig. 5B). Thus, cells displayed a high responsive state to N-pathway activation during the first 75 minutes of life instead of during the first 60 minutes as in control conditions. This period was followed by a state of low responsiveness with the period of transition ranging between 75 to 90 minutes after birth. (p<0.05, Chi-square Test and Bonferroni correction). Furthermore, the only significant difference between the N-response under Dap-overexpression conditions and that in control conditions was obtained with heat shocks delivered between 75 to 90 minutes after pI division (29% versus 68% in neu>dap conditions, p-value<0.0008, Fisher's Exact Test). This shows that the enhanced response window to N-signalling was shifted in time to accompany the delay in the S-phase.

These results were complemented by data obtained from pupae heterozygous for *brahma (brm)*, a gene that encodes a subunit of a complex that maintains transcriptional activation patterns by remodelling chromatin structure [29]. BrdU staining indicated that the onset of the S-phase in these pupae was not affected but that the S-phase was prolonged by about 30 minutes (Fig. 5A, *neu/brm^2^*). Under these conditions, we observed a shift in the response to N-activation as revealed by the significantly enhanced response to N-activation between 75 to 90 minutes after pI division (p-value<0.004, Fisher's exact Test, Fig. 5C). The fact that the timing of the cellular response to N-activation changes accordingly with changes in the timing of the S-phase confirms the idea that the enhanced sensitivity to N-activation is related to the S-phase.

### Ttk expression was inhibited after S-phase abolition

**Figure 6:**
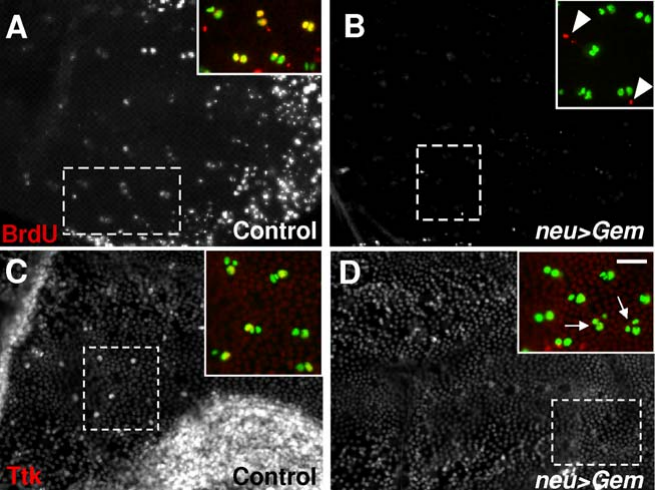
S-phase suppression decreased *ttk* gene expression. Low magnification views of nota in *neu>H2B::YFP* pupae in A and C and *neu>H2B::YFP,Gem* pupae in B and D. The incorporation of BrdU is shown in frames A and B while the expression of *ttk* is shown in C and D. The dotted squares denote the region that is detailed in the respective inset in which sensory cells are shown in green and BrdU or Ttk in red. BrdU incorporation was completely abolished in secondary precursor cells after specific ectopic expression of Gem (compare A and B) but not in epidermal cells (arrowheads in the inset of B). Note that Ttk expression was considerably reduced after specific ectopic expression of Gem (compare C and D). Arrows in D indicate three-cell sensory organs. Pupae at 19 h APF. Conditional expression of Gem was performed using the Gal4/ Gal80^ts^ system. Scale bar: 20 µm.

In order to confirm that cell responsiveness to N-activation is enhanced during the S-phase, we blocked the onset of the S-phase by overexpressing *Geminin (gem)*. Gem is a licensing replication factor that prevents DNA re-replication by binding to Dup/Cdt1 [30], [31]. Conditional expression of *gem* was performed using the Gal4/Gal80^ts^ system. In control sensory organs at 18 h APF (two-cell stage), 88% of the cells were BrdU positive (yellow cells in Fig. 5A). Furthermore, several epidermal cells positive to BrdU were also observed (red cells in the inset of Fig. 6A). At a similar stage, no BrdU incorporation was observed in sensory cells when *gem* was specifically expressed in the bristle lineage (Fig. 6B). The fact that we observed BrdU positive epidermal cells confirmed that the S-phase was specifically abolished in the bristle lineage (arrowheads in the inset of Fig. 6B). Under these conditions, we analysed cell responsiveness to N-activation by testing the expression of the fate determinant Tramtrack. Control clusters at 19h APF were composed mainly of two cells (92%), one of them being Ttk-positive (Fig. 6C). In contrast, Ttk was undetectable in two-cell clusters when Gem was overexpressed. This absence in Ttk expression was observed even in late clusters in which some cells had already entered the next round of mitosis (arrows in Fig. 6D). The absence of *ttk* expression indicates that N-pathway activity was impaired when the S-phase was abolished.

### HP1 blocks the residual response to N-activation during the G2-phase

During the S-phase, chromatin undergoes major structural modifications that are inherent to DNA replication [32]. We investigated whether the heightened cell receptivity to N-activation during the S-phase is due to these changes in chromatin structure. The cellular response to N-activation was analysed under several conditions known to modify chromatin structure packaging. These conditions included *osa^eld309^* mutants as well as overexpression of a dominant negative form of Brahma (*brm^K804R^*) or Osa. In all these cases, the timing of the N-response was modified (not shown). Unfortunately, the timing of the S-phase was also modified under these same conditions. This prevented us from clearly separating the effect of changes in chromatin structure from the effect of changes in S-phase timing on N-signalling receptivity.

**Figure 7:**
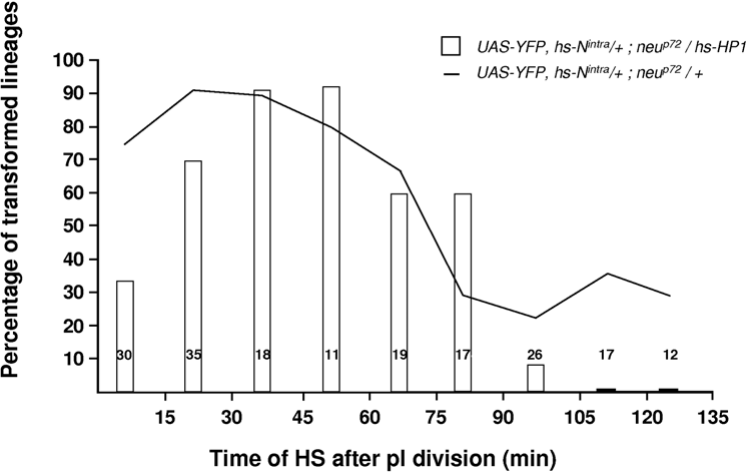
Overexpression of the Heterochromatin Protein-1, HP1, blocks the late residual cellular response to the N pathway. Probability of obtaining transformed sensory organs (resulting from the formation of two pIIa cells) plotted against the moment of overexpression of both N^intra^ and HP1 (*hs>N^intra^*, *HP1* pupae). For comparison, the control data (*hs-N^intra^* pupae alone) are presented as a continuous line. These control data are the same as those shown in Fig. 5. Note that when HP1 was expressed late in the G2-phase (between 105 and 135 min), cells became insensitive to N-pathway activation. The HS was applied under visual control in pupae at 19 h APF.

This ambiguity was clarified by experiments involving the overexpression of Heterochromatin Protein 1 (HP1). This factor recognizes methylated tags in histoneH3 and triggers chromatin-mediated gene silencing, probably inducing high-order chromatin structures that propagate along the chromosome [33]. We used *hs-HP1* and *hs-N^intra^* constructions to ectopically express *HP1* together with *N^intra^* at different moments of the secondary precursor cell life in living pupae. We observed that the cell response to N-activation obtained for heat-shocks delivered between 15 and 75 minutes after birth was unaffected when compared to control pupae (Fig. 7). A significant reduction in this response was observed for HS between 0 and 15 min APF as compared to control (p<0.0004, Fisher's Exact Test). This was probably due to some effect of HP1 on the onset of the S-phase. More interestingly, HP1 overexpression completely impaired cell responses to N-activation when the N-pathway was activated during G2 (between 105 and 135 min after pI division, Fig. 7).

The absence of N-response in G2 after HP1 overexpression suggests that N-responsiveness is modulated by modifications in the chromatin structure. Taken together, our data suggest that a “decompacted” chromatin state associated with the progression through the S-phase is responsible for the heightened sensitivity of cells to N-activation during S-phase.

## Discussion

We report here that secondary precursor cells of the bristle lineage are more responsive to N-signalling during the S-phase than during the G2-phase. These results show that cell responsiveness to signalling pathways, in particular to the N-pathway, fluctuates along the cell's life. Our findings highlight a fascinating aspect of cell dynamics in as much as the cellular response depends on the cell's competence level rather than on an instructive signal.

We used two independent thermo-sensitive conditions to activate the N-pathway at different times in the cell cycle of the secondary precursor cells: a temperature step from 30°C to 18°C using the thermo-sensitive hypomorph allele *N^ts-1^* and a temperature pulse from 25°C to 37°C using the thermo-inducible construct *hs-N^intra^*. The fact that cell responsiveness is enhanced in the S-phase is supported by the finding that (1) the period of enhanced sensitivity to the N-pathway coincided with the period of the S-phase; (2) a change in the timing of the S-phase resulted in a corresponding change in the timing of the N-response, and (3) when the S-phase was abolished, the N downstream target gene *ttk* failed to be expressed. In addition we present evidence suggesting that the Notch cellular response is sensitive to the “chromatin language” which regulates transcription.

### The cellular response to Notch depends on the moment of the cell cycle when the N-pathway is activated

Our data show that in an *N^ts-1^* background, the inactivation of the N-receptor at 30°C is a rapid process that takes place in less than 15 minutes. The *N^ts-1^* allele is a point mutation in one EGF repeat of the extracellular domain of the N-receptor and neither the transcription nor the translation are affected by this allele [34]. To date, the mechanisms underlying this thermo-sensitivity is unknown. The fact that the *N^ts-1^*-receptor is inactivated rapidly at 30°C suggests that the molecular conformation of the receptor is impaired in this allele. However, we cannot rule out the possibility that in this allele, N-receptor recycling is impaired. This rapid inactivation allowed us to maintain pupae at 18°C between 0 to 15 h APF to avoid a neurogenic phenotype and assured a rapid N-off condition when shifted to 30°C. The recovery of the N-function after a shift from 30°C to 18°C lasts for thirty minutes or less. We never observed a N-response in posterior secondary precursor cells when the temperature shift was applied during the last thirty minutes of life. It is probable that this response time is related in part to the recovery of N-receptor function, the time required to implement the cell response and to a particularly low level of cell responsiveness to the N-pathway during the last hour of life.

Following the HS-protocol, even short duration temperature pulses, such as those used in this study, provoked a transitory delay in pupal development as compared with control pupae maintained at 25°C. This delay could be extended for up to two hours, after which the time-curse of development was restored to normal. During this general retard, heat shocks selectively induced transcription of heat shock-controlled mRNA [35], [36]. In particular, N^intra^ is detectable in the nuclei 20 minutes after HS independently of whether the HS was applied in the S- or G2-phase. Furthermore, we observed that Ttk was ectopically expressed 60 min following HS. As a consequence, given the rapid accumulation of N^intra^ (within 20 minutes) and the global developmental delay induced by the heat-shock, the activation of the N-pathway by the overexpresssion of N^intra^ is probably concomitant to the cell cycle phase at the moment of the heat-shock. Thus, in the *HS-N^intra^* construction, cells have enough time to elaborate their response when the N-pathway was directly activated during the S- or the G2-phases.

These considerations, together with the lack of correlation between the probability of acquiring an N-on identity and the time-lapse following N-activation (see Fig. 4), show that the fact that cells respond less well to N-activation during the G2-phase is not due to time constraints related to the implementation of a cell-response. Rather, cells appear to have different sensitivity to Notch when the N-pathway is activated in the S- or in the G2-phases.

### Abolition of the S-phase impaired ttk expression

A dramatic reduction in *ttk* expression was observed when the S-phase was abolished after overexpression of the licensing replication factor, Gem. Since Gem overexpression was performed after pI specification, when cells are G2-arrested [37], the division of pI was normal. As such, the resulting two secondary precursor cells were genetically normal under these Gem-overexpression conditions. Thus, the dramatic reduction in Ttk expression observed in secondary precursor cells was not due to a problem of aneuploidism. Instead, this reduction seems to be related to the abolition of the S-phase itself.

Alternatively, the lack of Ttk expression may be due to a change in cell identity induced by Gem. It has been shown that, in *Xenopus* embryos, the number of neurons increased after overexpression of Gem. Thus, Gem seems to act directly as a neuralizing factor [38]. As such, the observed reduction in *ttk* expression after Gem overexpression can be interpreted as a premature neuronal transformation of secondary precursor cells. We think that this possibility is unlikely because we never observed neuronal markers such as Elav in secondary precursor cells that would suggest a cell transformation. Cell death, probably due to generalized aneuploidy following secondary precursor cell divisions, prevents us from performing a detailed analysis of the identity of S-phase arrested cells. In any case, we cannot rule out a potential neuralizing effect of Gem to explain our results, neither can we eliminate the possibility that the neuralizing action of Gem suggested by previous work results from a failure of non-neuronal fate determinant pathways to act after S-phase arrest.

Apart from its effect on chromatin remodelling, it has been suggested that Brm can also have a direct action on cell identity. Loss of function of *brm* results in double shaft-double socket sensory organs [39], a phenotype that resembles those observed in Notch gain of function conditions [40]. However, a more recent study shows, that under the same conditions, sensory organs present more than one neurone, a phenotype similar to that resulting after reduced Notch signalling [41]. The different experimental conditions used in these two studies can explain this discrepancy. Thus, a fine-tuning of *brm* function can result in opposite phenotypes suggesting that the action of Brm on the Notch pathway is more complex than that of a cell fate modulator. This complexity can be due to the change in the cell responsiveness to Notch due to cell cycle phase modifications as revealed in the present work.

Taken together, our data show that the cell sensitivity to N-activation depends on the progression through the S-phase. It is important to state that, although we show that DNA synthesis is critical for *ttk* expression, we cannot extend these observations to the expression of all N-target genes. Given the complexity of the transcriptional complex in which N receptor is involved, one would expect that all N-target genes are not restricted to the same degree of dependence on DNA-replication conditions. Systematic analysis of the expression of N-target genes is required to resolve this problem.

### Cells responsiveness in G2-phase

During the G2-phase, the response to N^intra^ was reduced but not completely abolished. We show that about 30% of cells responded when N was activated at different moments during the G2-phase. A response to N-activation during this phase is compatible with previous observations showing that N-mediated lateral inhibition occurs among proneural cells arrested in the G2-phase [42]–[44]. However, the sensitivity of cells to N-activation as a function of the cell cycle could be different in quiescent cells (cells during sensory precursor cell selection involving lateral inhibition) from that in mitotically active cells (cells in the bristle lineage). In fact, the molecular mechanisms involved in N-signalling may be different in these cell types. This is indeed the case for different cells within the bristle lineage itself. For example, while the N pathway is required for the determination of both outer and inner cells, Su(H) is essential only for the determination of the former [45]. By analogy, one must be cautious when comparing cell responsiveness to N-signalling as a function of cell cycle phases in quiescent and mitotically active cells.

### DNA replication, chromatin assembly and Notch target gene transcription

The enhanced sensitivity to N-activation during the S-phase can be ascribed to either an association of N-signal transductor factors with the DNA replication machinery or a specific availability of N-signal transductor factors during the S-phase. Alternatively, a local decompacted chromatin state during S-phase can favour the trans-acting factor recruitment on N target genes. We favour this last hypothesis since deregulation of chromatin packing affected the response to N-signalling. Increasing evidence indicates that higher-order chromatin compaction presents an efficient barrier to DNA trans-acting factors [46]. We observed that HP1 overexpression abolished the residual N-dependant response occurring during the G2-phase. Although HP1 has been originally identified as a heterochromatin-associated protein, its function in regulation of euchromatic gene transcription is beginning to be elucidated [47]. In fact, HP1 associates with approximately 200 euchromatic sites on *Drosophila* polytene chromosomes [48] and an increasing number of reports show that HP1 regulates gene expression during cell differentiation [49]. As such, HP1 overexpression may induce a local “closed” chromatin structure rendering it inaccessible to transcription factors that promote some of the N target genes. In addition, it has been shown that tethering HP1 to euchromatic sites of *Drosophila* chromosomes is sufficient to nucleate a silent chromatin conformation in the promoter region of genes whose transcriptional activity is low or absent [50]. Thus, the formation of a closed chromatin structure will be facilitated all the more when cells are in the G2-phase, a phase in which we show a reduced cell responsiveness to N-activation. In agreement with this possibility, HP1 overexpression did not affect the cell responsiveness to N-activation in the S-phase where N-target genes seem to be set to be transcribed. Furthermore, this lack of effect when HP1 was applied during the S-phase rules out the possibility that HP1 has cell-cycle independent actions on transcription.

### Concluding remarks

This work shows that the N-induced response in cells of the bristle lineage is enhanced during the S-phase. We suggest that high-order chromatin structures associated with the progression through the S-phase create favourable conditions for N-target gene transcription. We highlight a new mechanism that regulates N-target gene expression during cell fate acquisition. This mechanism couples DNA synthesis and N signalling to reprogram the default state (N-off, pIIb identity) to another (N-on, pIIa identity). The enhanced sensitivity of cells to respond to cell-fate signalling during the S-phase seems to be a widespread phenomenon. For example, progression through the S-phase is required for the expression of *even-skipped* during specification of cell identity in the central nervous system of *Drosophila* [8]. In addition, the S-phase is clearly important during de-differentiation of tobacco protoplasts [51]. Moreover, vulva-precursor cells in *C. elegans* seem to be more sensitive to N-signaling in late G1- or early S-phases [52]. We do not know whether this sensitivity is a general property of the S-phase or is a local characteristic that affects a restricted number of genes. Further experiments are required to address this issue. In this regard, it will be of interest to know whether the expression of other N-target genes or even the signalling efficiency of other pathways also depend on the progression through the S-phase.

## Materials and Methods

### *Drosophila* stocks

The neuralized^P72^-Gal4 (*neur>*) line was used to express the constructions *UAS-histone2B::YFP (H2B::YFP)* [20]; *UAS-Dacapo (UAS-Dap)* [28] and *UAS-Geminin* (a gift from H. Richardson) in the bristle lineage using the Gal4/UAS expression system [21]. In some experiments, the *UAS-Pon::GFP* construction was used to distinguish the polarity of cell divisions [43]. The H2B::YFP construction that highlights the DNA was used to track cells of the lineage during *in vivo* imaging. The combination *UAS-H2B::YFP;Tubulin-Gal80^ts^,neur^p72^Gal4/SM5CyO-TM6Tb* was used to evoke a conditional expression of *UAS-Gem*. Gal80^ts^ is a thermosensitive version of the Gal4 repressor Gal80. In Gal80^ts^ background, Gal4 is repressed at 18°C and is fully active at 30°C. To overexpress Gem in bristle lineage cells, pupae were shifted from 18° to 30°C at 0h00 after pupal formation (APF). Overexpression of the active form of the Notch receptor (N^intra^) [53] and HP1 [54] was performed using heat shock constructs. The *brm^2^* is an amorph allele [55]. The *brm^K804R^* is a dominant negative allele [39]. The *N^ts-1^* is a temperature sensitive allele [34]. The developmental time at 30°C was similar to that at 25°C.

### *In vivo* imaging

Live imaging was carried out using a spinning disc coupled to an Olympus BX-41 microscope (Ropert Scientific, France) (20×, NA 0,5 or 40×, NA 0,75 objective, CoolSnapHQ^2^ camera) driven by Metamorph software (Universal Imaging). Z-stacks of images were acquired every 3–6 minutes and assembled using ImageJ software. Pupae dissection was carried out as described previously [13]. The temperature of the recording chamber was carefully controlled (±0.1°C) using a home-made temperature controller containing a Peltier device fixed to the microscope stage. During *in vivo* experiments, the endogenous Notch pathway was able to be activated in the *N^ts-1^* line by a temperature shift from 30°C to 18°C. In order to circumvent a neurogenic phenotype due to the early loss of N function [12], *N^ts-1^* pupae were maintained at 18°C between 0 and 15 h APF and shifted to 30°C afterwards. This initial N-off condition was assured by the fact that the N-function is rapidly inactivated after passage to restrictive temperature (se Supporting data Fig. S1A). The developmental time at 18°C was twice longer than that at 25°C.

The N-pathway was overactivated using the N^intra^ line after a heat shock of 37°C for 10 min. Otherwise N^intra^ pupae were maintained at 25°C. Heat shocks caused a variable delay in development time of about 60 min (in some cases it could be extend until two hours). All analysis was centred on the two secondary precursor cells resulting from the division of the primary precursor cell. Sensory clusters in rows 1 to 4 were considered for statistical analysis. During *in vivo* recordings, the size of the nucleus (bigger for pIIa than for pIIb daughter cell), the number of rounds of subsequent cell divisions (one and two for pIIa and pIIb cells respectively) and the orientation of the divisions (planar and apico-basal for pIIa and pIIb cells respectively) were used as criteria to identify the resulting cells. After completion of the cell lineages (about 8 hours later at 18°C), the notum was dissected out and immuno-labelling with specific markers was performed to confirm the information obtained by *in vivo* observations.

### Immunohistology

Dissected nota were processed as described previously [14]. For combined time-lapse immunodetection imaging, the sensory organs analysed *in vivo* were unambiguously recognized in the fixed nota by their relative position with respect to landmarks such as the midline, the position of the macrochaetes or the rows of microchaetes. The following primary antibodies were used: mouse anti-BrdU (Becton Dickinson 1∶50); rabbit anti-GFP (Santa-Cruz, 1∶500); rabbit anti-Ttk (gift from A. Travers, 1∶500); mouse anti-Elav (DHSB, 1∶100); rat anti-Su(H) (gift from F. Schweisguth, 1∶500); mouse anti-Repo (gift from B. Jones); mouse anti-Notch (DHSB, C17-9C6, 1∶500). Alexa 488- and 568-conjugated secondary antibodies anti-mouse, anti-rat and anti-rabbit were purchased from Molecular Probes and used at 1∶1000. Cy5 conjugated antibodies anti-mouse, anti-rat or anti–rabbit were provided from Promega and were used at 1∶1200. S-phase characterisation using BrdU incorporation was performed as described [19]. Images were processed with ImageJ software and Adobe Photoshop software.

## Supporting Information

Figure S1 Activation and inactivation kinetics of the *N^ts-1^* allele. (A) Kinetics of *N^ts-1^* allele inactivation. Temperature shifts from 18°C (permissive temperature) to 30°C (restrictive temperature) were applied at different times before and after pI division. Inactivation of the N-receptor was confirmed by the formation of organs composed exclusively of inner cells (transformed lineages in which both secondary precursor cells acquired a N-off pIIb identity, ii). The percentage of transformed sensory organs is plotted as a function of the time of the temperature shift around the pI division (abscissa). Note that posterior secondary cells did not implement a N-response for temperature shifts during the first 15 minutes after birth (pI division). This suggests that *N^ts-1^* receptors become non-functional after only 15 minutes at 30°C. (B) Kinetics of *N^ts-1^* allele activation. Temperature pulses of variable duration to 18°C were applied to *N^ts-1^* pupae maintained at 30°C. These pulses were applied during the first 30 minutes after pI division. The formation of normal sensory organs (ii, in which the posterior cell acquired an N-on pIIa identity) was used as an index of N-activation. The proportion of normal sensory organs is plotted as a function of the length of the temperature pulse. Note that, for 30 min pulses, we observed a normal set of sensory cells in more than 50% of clusters analyzed. This shows that pulses of 30 minutes (which corresponds to 15 min at 25°C) were long enough to trigger a N-response. Temperature shifts were applied under visual control under time-lapse imaging conditions in *N^ts-1^/Y; neu>H2B::YFP* pupae.
(0.21 MB TIF)

Figure S2 Kinetics of the *N^intra^* activation. (A) *HS-N^intra^ neu>H2B::YFP* pupae were imaged in vivo and a heat-shock (10 min at 37°C) was delivered at different times after pI division. Using specific antibodies against the intracellular domain of the N-receptor, the ectopic expression of *N^intra^* in the anterior secondary precursor cell was monitored at different times after HS. The plot shows the percentage of anterior cells in which the level of N^intra^ detected was above the level of that in epithelial cells. Black bars correspond to HSs delivered during the first hour after pI division. Empty bars correspond to HS delivered during the second hour of life. The bottom panels show representative examples of N^intra^ immunodetection in two-cell clusters at the period indicated. In each case, the upper image pair (Filled squares) corresponds to a HS delivered during the first hour after pI division and the bottom image pair (Empty squares) during the second hour of life. In each image pair, N^intra^ immunoreactivity is shown alone on the left and in red on the right (merge). YFP is in green. Doted lines in the N^intra^ panels delimit the nuclei. In each image, anterior is on the top. The endogenous level of N^intra^ was not detected (see panels at 5 min). Note that N^intra^ was detected 20 min after HS independently of the time at which the HS was applied. Ninety minutes after HS, the immunolabeling was indistinguishable from the background in more than 80% of cluster analyzed. The number of two-cell clusters analyzed is indicated within each bar. Scale bar: 5 µm.
(1.16 MB TIF)

Figure S3 Cells are more receptive to endogenous N-pathway activation during their first hour of life. Representative frames of two microchaete lineages from an *N^ts^/Y; neu>H2B::YFP* pupae imaged in vivo followed by immunostaining. The temperature shift was applied at 18h54 after pupal formation (APF). Brackets indicate cell divisions. Anterior is on the right. Time APF is shown at the bottom left of each frame. At 18°C, development proceeds half as quickly as at 25°C. In the last frame, bristle lineage cells are revealed by GFP antibodies (green). Ttk and Repo immunoreactivities were used to identify outer cells (socket, so, and shaft, sh, yellow/red and the glial, g, blue) cells respectively. The other cells were identified by their characteristic divisions recorded *in vivo*, n: neuron, st: sheath. Each image results from the merge of 5 horizontal optical sections. Scale bar: 10 µm.
(0.54 MB TIF)

Figure S4 Overexpression of N-pathway during the first 45 min of life induces an ectopic expression of Tramtrack in the anterior cell. The expression of Ttk was used as an index of the Notch pathway activation in the anterior secondary precursor cell. Combined in vivo recording and immunodetection of *neu>H2B::YFP HS-N^intra^* pupae which were heat-shocked at different times after pI division (in minutes, indicated in the upper left corner). Immunodetection was performed at least 90 min after HS application to allow the cells to recuperate. Secondary precursor cells were identified by anti-GFP immuno-reactivity (green), Ttk detection is in red (yellow). Anterior cell is on the top. In the control situation, Ttk is found only in the posterior pIIa cell. Note that the anterior cell ectopically accumulated Ttk only when the N^intra^ HS pulse was applied between 0 and 45 min after pI division. Scale bar: 5 µm.
(1.55 MB TIF)

Figure S5 Numb blocks the N-response when was ectopically expressed within the first 30 min after pI division. A *H2B::YFP/HS-numb, neu>Gal4* pupae was heat shocked at a given time after pI division under time-lapse conditions. The formation of sensory organs composed exclusively of inner cells revealed the blockade of the normal N-response in the posterior secondary precursor cell (transformed lineage, ii). The plot shows the percentage of transformed lineages as a function of the time of HS application. Note that the overexpression of Numb effectively blocks the N-response only when the HS was applied during the first 30 minutes of life.
(0.16 MB TIF)

Movie S1 *In vivo* observation of a *N^ts-1^; neur> H2B::YFP* pupa showed in Fig 2. Anterior is on the left and the view is dorsal. The colour bar represents the temperature: red when pupae were maintained at 30°C and blue when the temperature was dropped to 18°C. Time (h:min APF) is shown at the top left. Each image results from the merge of 5 horizontal optical sections.
(0.40 MB MOV)

Movie S2 *In vivo* observation of a in *HS-N^intra^; neu>PON::GFP, H2B::YFP* pupa showed in Fig 3. Anterior is on the top and the view is dorsal. The red bar around 18h18 APF represents the heat shock to 37°C for 10 min. Otherwise pupae were maintained at 25°C. Time (h:min APF) is shown at the top left in each frame. Each frame results from the merge of 7 horizontal optical sections.
(0.47 MB MOV)
