# Supplementary figures and images for: S-Phase Favours Notch Cell Responsiveness in the Drosophila Bristle Lineage
Source: PLoS One. 2008 Nov 5;3(11):e3646. doi: 10.1371/journal.pone.0003646 (PMC2574411; doi:10.1371/journal.pone.0003646)

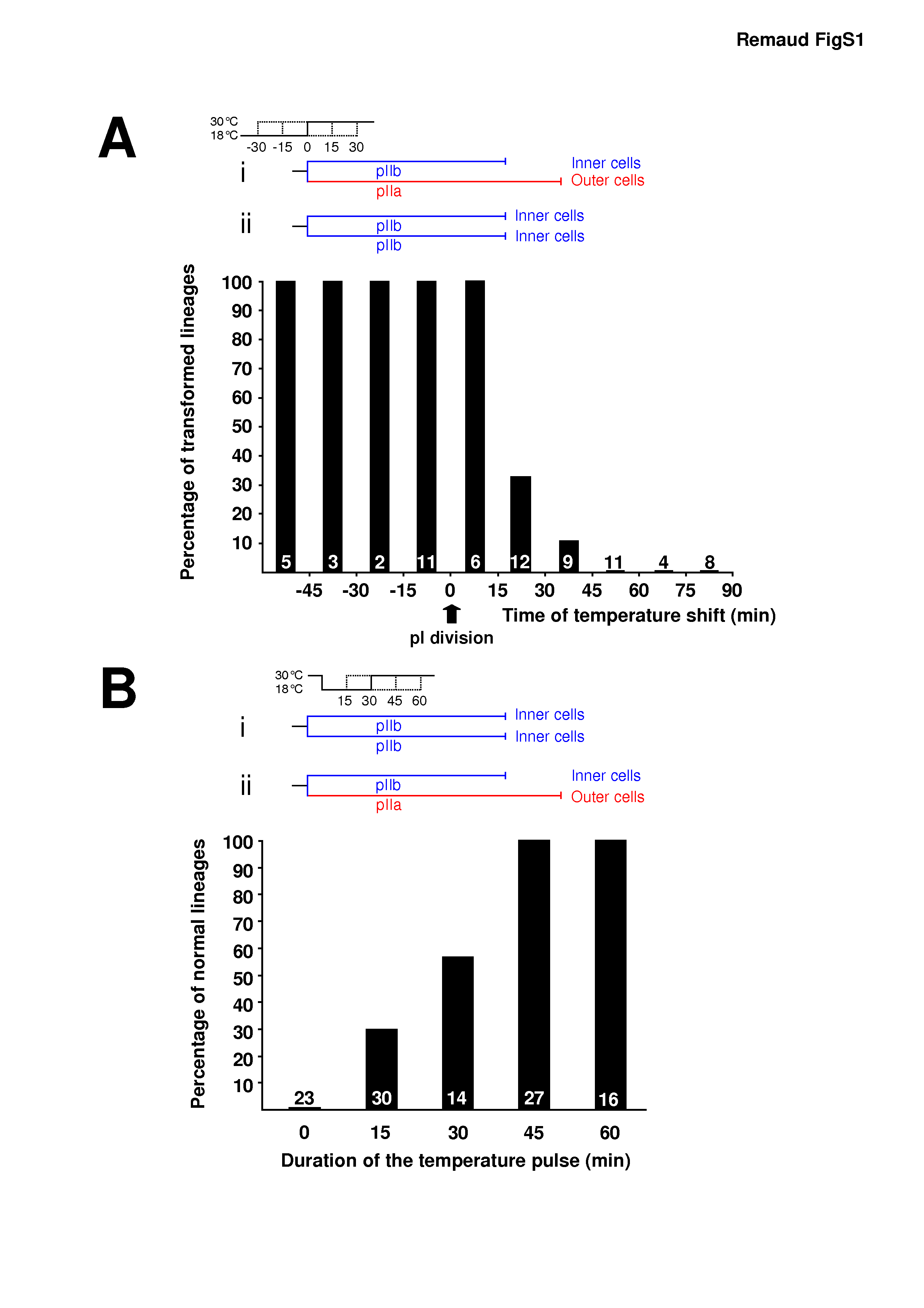

Supplement: Figure S1 — Activation and inactivation kinetics of the Nts-1 allele. (A) Kinetics of Nts-1 allele inactivation. Temperature shifts from 18°C (permissive temperature) to 30°C (restrictive temperature) were applied at different times before and after pI division. Inactivation of the N-receptor was confirmed by the formation of organs composed exclusively of inner cells (transformed lineages in which both secondary precursor cells acquired a N-off pIIb identity, ii). The percentage of transformed sensory organs is plotted as a function of the time of the temperature shift around the pI division (abscissa). Note that posterior secondary cells did not implement a N-response for temperature shifts during the first 15 minutes after birth (pI division). This suggests that Nts-1 receptors become non-functional after only 15 minutes at 30°C. (B) Kinetics of Nts-1 allele activation. Temperature pulses of variable duration to 18°C were applied to Nts-1 pupae maintained at 30°C. These pulses were applied during the first 30 minutes after pI division. The formation of normal sensory organs (ii, in which the posterior cell acquired an N-on pIIa identity) was used as an index of N-activation. The proportion of normal sensory organs is plotted as a function of the length of the temperature pulse. Note that, for 30 min pulses, we observed a normal set of sensory cells in more than 50% of clusters analyzed. This shows that pulses of 30 minutes (which corresponds to 15 min at 25°C) were long enough to trigger a N-response. Temperature shifts were applied under visual control under time-lapse imaging conditions in Nts-1/Y; neu>H2B::YFP pupae. (0.21 MB TIF) [file pone.0003646.s001.tif]

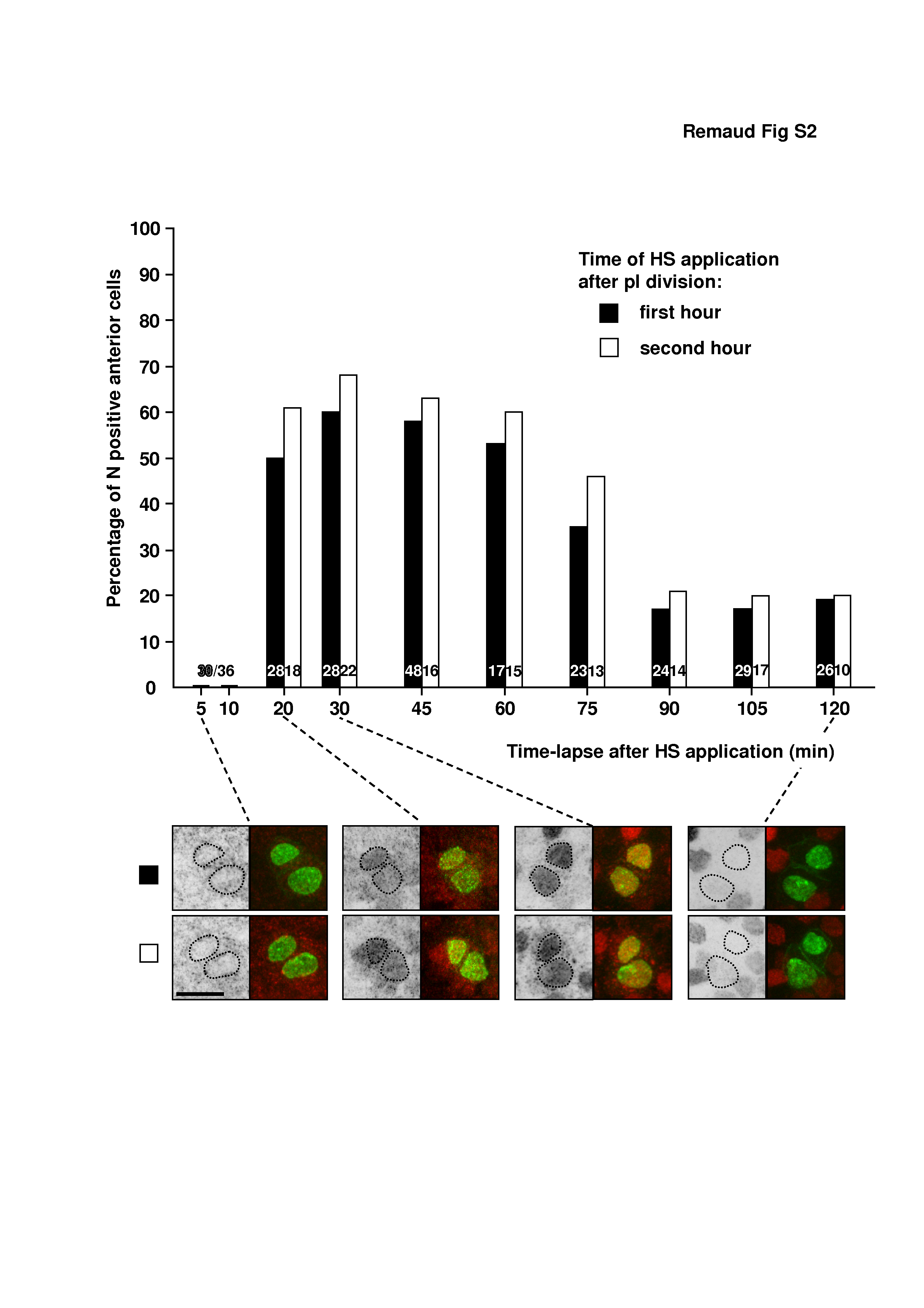

Supplement: Figure S2 — Kinetics of the Nintra activation. (A) HS-Nintra neu>H2B::YFP pupae were imaged in vivo and a heat-shock (10 min at 37°C) was delivered at different times after pI division. Using specific antibodies against the intracellular domain of the N-receptor, the ectopic expression of Nintra in the anterior secondary precursor cell was monitored at different times after HS. The plot shows the percentage of anterior cells in which the level of Nintra detected was above the level of that in epithelial cells. Black bars correspond to HSs delivered during the first hour after pI division. Empty bars correspond to HS delivered during the second hour of life. The bottom panels show representative examples of Nintra immunodetection in two-cell clusters at the period indicated. In each case, the upper image pair (Filled squares) corresponds to a HS delivered during the first hour after pI division and the bottom image pair (Empty squares) during the second hour of life. In each image pair, Nintra immunoreactivity is shown alone on the left and in red on the right (merge). YFP is in green. Doted lines in the Nintra panels delimit the nuclei. In each image, anterior is on the top. The endogenous level of Nintra was not detected (see panels at 5 min). Note that Nintra was detected 20 min after HS independently of the time at which the HS was applied. Ninety minutes after HS, the immunolabeling was indistinguishable from the background in more than 80% of cluster analyzed. The number of two-cell clusters analyzed is indicated within each bar. Scale bar: 5 µm. (1.16 MB TIF) [file pone.0003646.s002.tif]

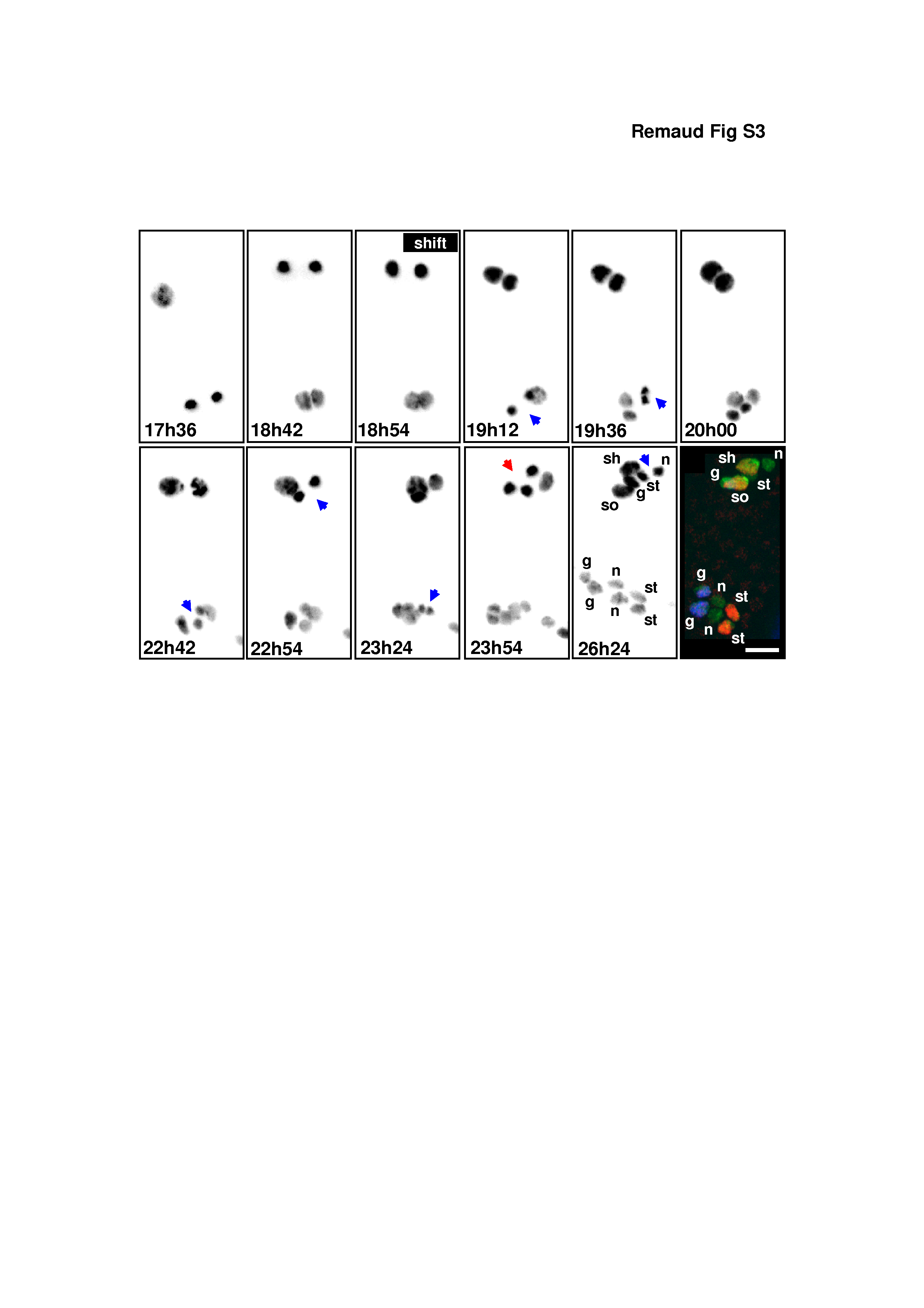

Supplement: Figure S3 — Cells are more receptive to endogenous N-pathway activation during their first hour of life. Representative frames of two microchaete lineages from an Nts/Y; neu>H2B::YFP pupae imaged in vivo followed by immunostaining. The temperature shift was applied at 18h54 after pupal formation (APF). Brackets indicate cell divisions. Anterior is on the right. Time APF is shown at the bottom left of each frame. At 18°C, development proceeds half as quickly as at 25°C. In the last frame, bristle lineage cells are revealed by GFP antibodies (green). Ttk and Repo immunoreactivities were used to identify outer cells (socket, so, and shaft, sh, yellow/red and the glial, g, blue) cells respectively. The other cells were identified by their characteristic divisions recorded in vivo, n: neuron, st: sheath. Each image results from the merge of 5 horizontal optical sections. Scale bar: 10 µm. (0.54 MB TIF) [file pone.0003646.s003.tif]

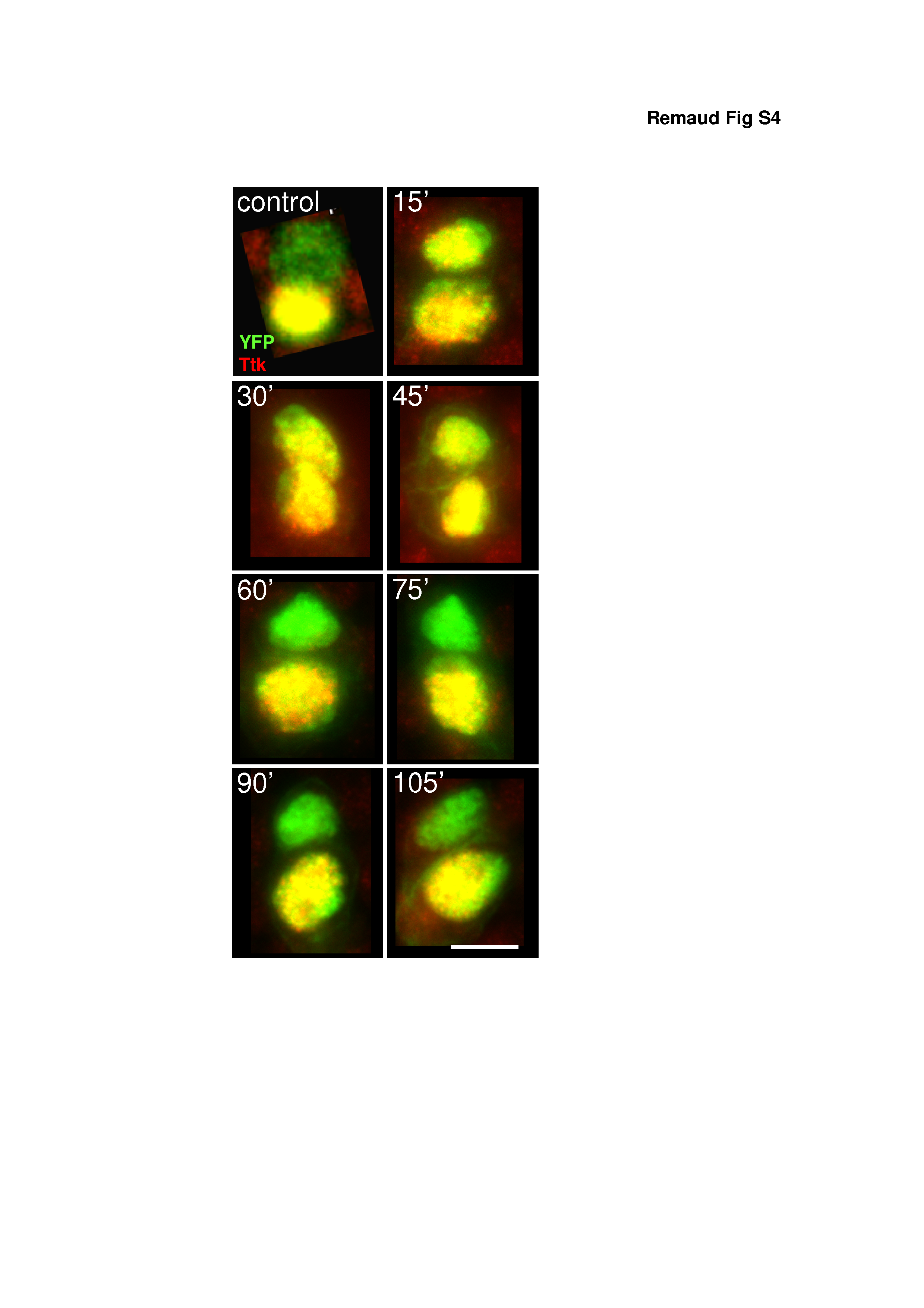

Supplement: Figure S4 — Overexpression of N-pathway during the first 45 min of life induces an ectopic expression of Tramtrack in the anterior cell. The expression of Ttk was used as an index of the Notch pathway activation in the anterior secondary precursor cell. Combined in vivo recording and immunodetection of neu>H2B::YFP HS-Nintra pupae which were heat-shocked at different times after pI division (in minutes, indicated in the upper left corner). Immunodetection was performed at least 90 min after HS application to allow the cells to recuperate. Secondary precursor cells were identified by anti-GFP immuno-reactivity (green), Ttk detection is in red (yellow). Anterior cell is on the top. In the control situation, Ttk is found only in the posterior pIIa cell. Note that the anterior cell ectopically accumulated Ttk only when the Nintra HS pulse was applied between 0 and 45 min after pI division. Scale bar: 5 µm. (1.55 MB TIF) [file pone.0003646.s004.tif]

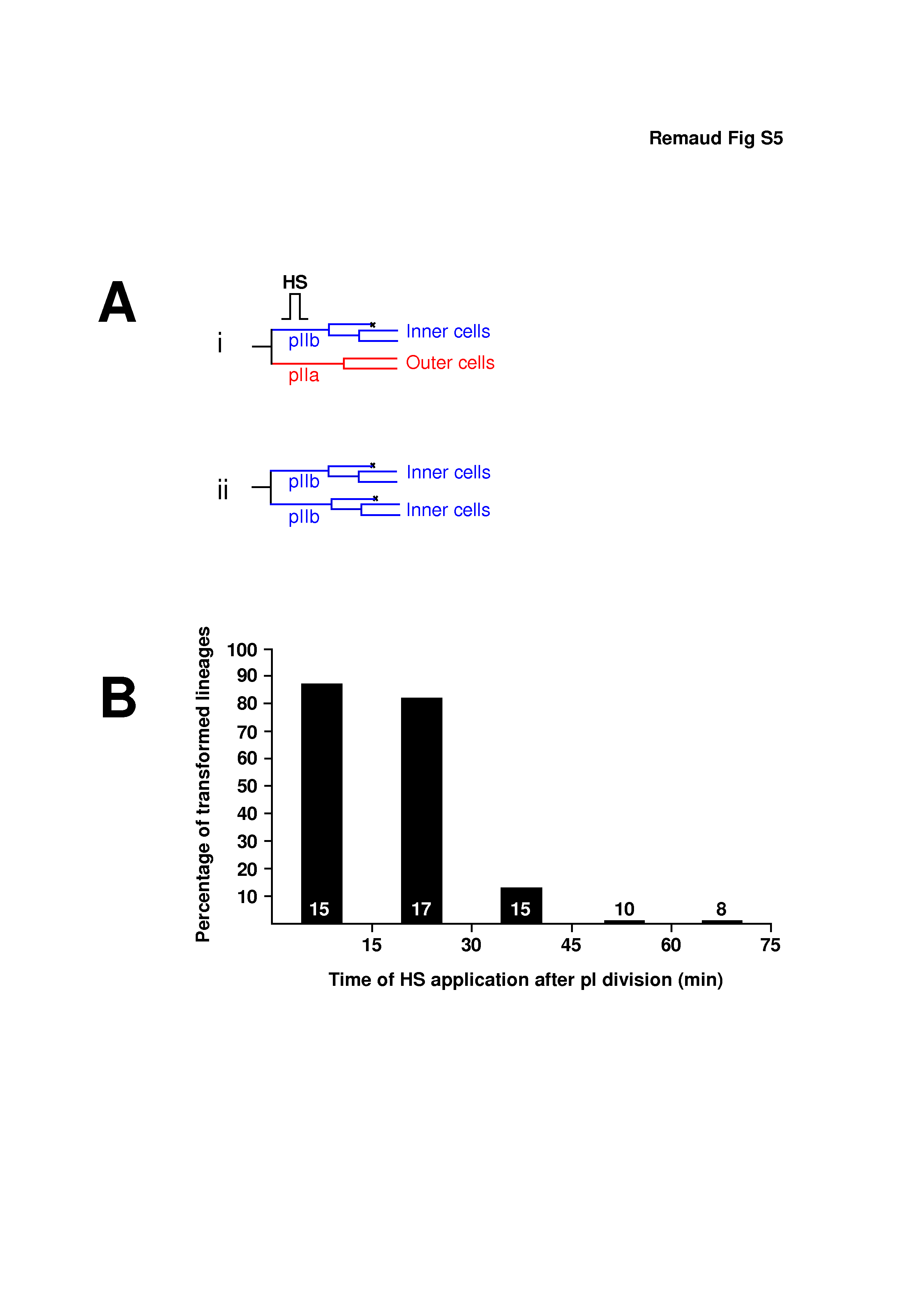

Supplement: Figure S5 — Numb blocks the N-response when was ectopically expressed within the first 30 min after pI division. A H2B::YFP/HS-numb, neu>Gal4 pupae was heat shocked at a given time after pI division under time-lapse conditions. The formation of sensory organs composed exclusively of inner cells revealed the blockade of the normal N-response in the posterior secondary precursor cell (transformed lineage, ii). The plot shows the percentage of transformed lineages as a function of the time of HS application. Note that the overexpression of Numb effectively blocks the N-response only when the HS was applied during the first 30 minutes of life. (0.16 MB TIF) [file pone.0003646.s005.tif]
